# “I wouldn’t have joined if it wasn’t online”: understanding older people’s engagement with teleyoga classes for fall prevention

**DOI:** 10.1186/s12906-022-03756-1

**Published:** 2022-11-02

**Authors:** Abby Haynes, Heidi Gilchrist, Juliana S Oliveira, Catherine Sherrington, Anne Tiedemann

**Affiliations:** 1 Institute for Musculoskeletal Health, The University of Sydney and Sydney Local Health District, Sydney, Gadigal Country, NSW Australia; 2 School of Public Health, Faculty of Medicine and Health, The University of Sydney, Gadigal Country, Sydney, NSW Australia

**Keywords:** Teleyoga, Fall prevention, Randomised controlled trial, Older people, process evaluation, Qualitative methods

## Abstract

**Background:**

Yoga-based exercise is a promising strategy for promoting healthy ageing, with the potential to reduce falls and increase physical, cognitive and psychological wellbeing. Teleyoga (real-time yoga provided via interactive videoconferencing) can deliver yoga programs at scale, potentially reducing costs, increasing convenience, and reaching people who cannot attend studio-based classes. But better understanding of how older people perceive and engage with teleyoga is needed to optimise its design, implementation and promotion.

**Methods:**

This study built on a previous realist process evaluation of the SAGE yoga trial which is testing the effect of a yoga-based exercise program on falls among 700 community-dwelling people aged 60 + years. In this second phase of evaluation we conducted focus groups with participants who had completed the SAGE program online and with the yoga instructors who were delivering it. We also conducted interviews with participants who had withdrawn from the trial. Six program theories developed in the earlier evaluation provided a framework for data analysis, supplemented by inductive coding and an analytical workshop.

**Results:**

Participants described physical and psychological benefits from the SAGE teleyoga program. While noting that teleyoga cannot facilitate hands-on correction or the same quality of observation or interaction as studio classes, participants were highly appreciative of their yoga instructors’ strategies for optimising visibility, instruction, social connection and therapeutic alliance, and for adapting to constrained home environments. Some participants argued that teleyoga was superior to studio classes due to its accessibility and convenience, its lower exposure to potential embarrassment about physical appearance or capabilities, and a reduced sense of peer competition and distraction. Our program theories applied across studio and online modes of delivery.

**Conclusion:**

Teleyoga increases accessibility for people in diverse locations and circumstances; it provides a psychologically safer space which combats self-consciousness and unwanted competitiveness; it may enhance embodiment and mindfulness for some; and it has the potential to be offered relatively cheaply at scale which could support free or reduced price classes for people on low incomes and pensions, thereby encouraging a wider population to engage in yoga for healthy ageing and fall prevention.

**Supplementary Information:**

The online version contains supplementary material available at 10.1186/s12906-022-03756-1.

## Introduction

Falls present serious problems for individuals and carers, health services and the economy [1–4]. People aged 60 and over have the highest risk of fall-related death or serious injury, and this risk increases with age [5]. There is strong evidence that exercise reduces the rate of falls and the number of older community-dwelling people who fall, especially programs involving balance and functional exercises [6]. To date, there is less evidence about yoga’s impact on falls, but yoga has demonstrable balance, mobility and physical function benefits for older people [7–9] and has been found to be acceptable as a fall prevention strategy, particularly for ‘younger’ and healthier people aged 60 and over [10]. There is also increasing evidence of yoga’s many other physical, cognitive and psychological health benefits [11–15], including efficacy in managing chronic pain [16–18], depression and anxiety [19, 20] and recovery from stroke [21, 22]: all conditions that commonly affect older people [23–26].

Teleyoga (real-time yoga classes provided via interactive videoconferencing [15]) is a promising way of delivering yoga programs at scale, potentially reducing costs, increasing convenience, and reaching people who cannot attend studio-based classes, including those affected by COVID-19 restrictions [27]. Teleyoga has been used with diverse populations to tackle various health conditions and has been found to be acceptable, with good adherence and positive health outcomes [28–32], equalling responses to in-person yoga programs [33]. Similarly, online fall prevention programs have been found to be feasible and acceptable to older participants [34, 35], supporting the premise of a teleyoga program designed to prevent falls. Although there is currently limited evidence about the effects of online exercise programs that offer synchronous (real time) versus asynchronous (pre-recorded) classes [36, 37], some studies suggest that synchronous programs are more engaging with higher levels of adherence and physical activity [38–40].

However, there are barriers to participating in teleyoga. While older people are increasingly engaged with digital technologies [41]—a trend that has been amplified by the COVID-19 pandemic [42]—the technology required to access teleyoga presents challenges [28, 29] which may be affected by disparities in age, socioeconomic status, education [42–44] and cultural background or context [45]. Attitudes to yoga in general can also present barriers; for example, beliefs about yoga worsening health problems or having negligible health benefits [46]. Lastly, factors that negatively influence attendance in other online physical activity programs may also impact teleyoga participation. These may include impractical home environments and lack of rapport with the program instructor [47]. Better understanding of how older people perceive and engage with teleyoga, and sustain yoga practice using online resources, can inform strategies for improved design, implementation and promotion of teleyoga and other online exercise programs [48, 49].

### The successful AGEing (SAGE) yoga trial

The Successful AGEing (SAGE) yoga trial is a randomised controlled trial testing the effect of a yoga-based exercise program on falls among 700 community-dwelling people aged 60 + years [50]. Participants are randomised to either:

1. The SAGE yoga program involving 40 weeks of supervised twice-weekly yoga-based exercise, plus goal-setting and unsupervised homework using specially designed yoga resources: videos and a handbook. The intervention is based on the Hatha/Iyengar style of yoga with longer static postures for standing poses designed to maximise balance, stability and leg strength to prevent falls. The program is easily modified to be accessible yet progressively challenging for different ability levels. It is run by experienced yoga instructors who are partners in the trial.
2. A seated yoga relaxation program taught over two group-based workshops and then undertaken individually. This program emphasises seated stretching and does not target standing balance.

The primary outcome is rate of falls in the 12 months following randomisation. Secondary outcomes include mental wellbeing, physical activity, health-related quality of life, balance confidence, physical function, pain, goal attainment and sleep quality. An economic analysis is comparing cost-effectiveness and cost-utility. Further details are available elsewhere [50].

Trial participants are recruited via local newspapers, community centres, seniors’ organisations and through social media. Exclusion criteria are: regular participation in yoga within the past 10 years; residence in a hostel or nursing home; presence of a medical condition that precludes exercise; cognitive impairment or limited English language skills.

The SAGE program was originally designed to be delivered face-to-face in yoga studios but, following public health responses to the COVID-19 pandemic, classes were moved online. Consequently, participants recruited early in the trial enrolled in studio-based classes but attended a ‘hybrid’ studio/online program, while those recruited later enrolled in and attended an exclusively online program, i.e. teleyoga. The transition to online classes was supported by the introduction of brief 1:1 Zoom meetings between each participant and instructor prior to the program, a WhatsApp group for each class and technology support from the research team. No type or size of device was specified for accessing online classes.

### Study aims

Our process evaluation sought to address three research questions:

1. In the SAGE yoga trial, what worked for whom, and under what circumstances?
2. What aided the transition from studio-based to online yoga classes?
3. How were different forms of program delivery valued by participants?

We conducted a previous realist evaluation to explain high levels of program adherence indicated in our routine process data and the largely favourable feedback participants gave about the hybrid program. This focused on questions 1 and 2 [51]. In this second phase of the process evaluation—the current study—we build on those findings to address question 3.

## Methods

### Building on work from phase one of the SAGE process evaluation

In phase one of the process evaluation we used a realist evaluation methodology to explore what worked for whom in the different program formats offered by the hybrid SAGE program. This is detailed in our methodology paper [52] but, in summary, it involved data collection across several sources, including interviews with 21 purposively sampled participants and three SAGE yoga instructors, and 46 participant feedback forms. We started by identifying the proximal impacts or process outcomes that we wanted to explain (such as strong adherence and positive self-reported impacts identified in feedback forms), and developed initial program theories to explain these. Then we collected data to test these theories and coded the data to highlight the suggested causal relationships that were most prominent. Throughout we drew on empirical literature to help us critique and refine our working theories and identify new theories that could explain emergent themes in the data. Table 1 provides an overview of the six program theories that resulted from this work and which answer research question one. This work is detailed elsewhere [51].

The current study sought to (a) further test these program theories with participants who had attended teleyoga classes exclusively (did these theories ‘hold’ or require revision?), and (b) explore participants’ and yoga instructors’ perceptions and experiences of online classes to help us increase.

our comparative understanding of how different forms of program delivery were valued by participants, and what conditions best supported positive engagement with teleyoga. We had previously found that the transition from face-to-face classes to online classes was aided by established relationships between participants and instructors, adaptations made by the instructors and participants’ gratitude at being able to access a structured “healthy” program during lockdown. However, post lockdown we continued to see high rates of recruitment and of program adherence over the 12 month teleyoga intervention with positive feedback form data from participants in relation to engagement, satisfaction and impacts on physical and psychological health.

**Table 1:** Overview of the results of phase one of the SAGE process evaluation

Program theories	Who did the interventions strategies work best for?	How did they work (mechanisms)?
1. It’s worth the effort	• SAGE attracted people who believed in the efficacy of yoga and who had interests in healthy ageing /or fall prevention • Experienced instructors encouraged everyone to practice at their own level, but it best suited those with physical capabilities in the moderate range who had manageable levels of pain	Value expectancy Therapeutic alliance Achievement/Mastery
2. In expert hands		
3. A communal experience	• Group classes suited those who valued social interaction and/or shared experiences • Studio-based classes suited those who liked to benchmark their physical competence and/or peer-check their poses	Shared experience Social connection Social comparison Position checking
4. Finding yoga within reach	• Free classes enabled people with financial constraints to try yoga and continue with it • Studio classes only worked for those with easy access to a participating yoga studio • Online classes required suitable home environments and willingness to use video conferencing software (Zoom) (with support)	Accessibility Convenience Gratitude
5. Building yoga habits	• Twice-weekly classes over 12 months plus homework suited people who could prioritise SAGE and were keen to progress • Flexible classes suited those with carer commitments, travel plans, injury or illness • Goal-setting was seldom adopted by participants in SAGE	Purposeful structure Momentum Accountability Continuity
6. Yoga’s special properties	• The SAGE program utilises traditional yoga practices which worked most effectively for those who were open to yoga as a disciplined and holistic practice	Embodiment Mindfulness

### Recruitment

For phase two of the process evaluation we had a sample frame of 49 people who had enrolled in teleyoga and completed SAGE exclusively online, and who had given prior permission to be contacted about taking part in qualitative evaluation. We invited 26 of these participants to take part in a focus group, recruiting purposively for maximum variation in age, perceptions of the program (as indicated by their final feedback form) and socioeconomic status across regional and metropolitan areas using postcodes as a proxy. We also sent email invitations to take part in an interview to nine participants who had dropped out of teleyoga classes and withdrawn from the trial but had given permission to be contacted about taking part in an interview. The four yoga instructors who are delivering the SAGE program and are partners in the trial were also invited to take part in a focus group.

### Data collection

Two researchers (HG and AH) facilitated three focus groups via videoconferencing software using a semi-structured question guide that focused on reasons for participation in SAGE, experiences of teleyoga and what conditions affected these experiences, and any impacts (Additional file 1). Due to the lower than expected rate of recruitment (n = 12) we anticipated running further participant focus groups; however, the views and experiences shared in these groups were so consistent both between the groups and in relation to our phase one findings, that we judged this to be unnecessary.

Interviews with withdrawn participants were conducted by one researcher (AH) by telephone and focused on reasons for participation, experiences of teleyoga, reasons for withdrawal and if anything could have been done to support them to continue with classes (Additional file 2). All trial participants gave informed consent via email.

The yoga instructors also took part in a focus group about their experiences of providing online classes and what teaching techniques they found best supported safe, effective and engaging teleyoga instruction (Additional file 3). This followed a workshop held with the whole research team (including the yoga instructors) in which we reviewed preliminary results of the process evaluation and sought the instructors’ expert views on them. Each focus group, and our research workshop, ended with an invitation to critique the six program theories (Table 1) which were shown on the screen using PowerPoint slides, illustrated with informal images.

### Data analysis

Audio recordings from focus groups and interviews were transcribed verbatim and uploaded to NVivo [53] where they were coded deductively using the framework of realist evaluation concepts and program theories developed in phase 1 of the process evaluation [52]. Eight inductive subcodes were introduced to capture new emphases in the data. Two researchers (HG and AH) independently reviewed a portion of the transcripts to determine this coding strategy, and one (HG) completed coding for the data set. The workshop described above was used to critique and refine emerging results.

## Results

Twelve SAGE participants took part in focus groups. Although 26 people were invited, eight declined or later withdrew due to ill health, caring commitments or travel, and six did not respond. Of the 12 focus group participants, nine were women, six lived in urban areas and six in regional or rural areas, four of which had low income status. Six were deemed to have a somewhat critical stance in relation to SAGE because they gave the program suboptimal ratings in their feedback forms. They ranged in age from 61 to 80 years with an average age of 67. Six ‘withdrawn’ participants responded to invitations to be interviewed but one of these declined and one did not return their consent form. The four withdrawn participants who took part in interviews were female and aged between 70 and 75, two were from high income urban areas and two from low or moderate income regional areas. The four SAGE yoga instructors were all female and had between 12 and 29 years of experience teaching yoga.

Our findings are now presented in relation to the six program theories outlined in Table 1. We focus on how well these theories and their mechanisms, identified in our previous process evaluation of the hybrid yoga program, apply to the post-COVID SAGE program where participants enrolled for and attended teleyoga, i.e. synchronous yoga classes delivered live and entirely online. Illustrative quotes are from that second phase of research. The age and gender of participants is included but quotes are otherwise deidentified, e.g. any names are pseudonyms.

### It’s worth the effort

Value expectancy (the extent to which people anticipate that benefits will outweigh costs [54]) was high at enrolment and sustained throughout the online program due to accessibility, high quality yoga instruction, the program’s progressive structure of twice-weekly classes, and the inherent characteristics of yoga (all explored below). Participants described many resulting health benefits which made yoga worth the effort. These included improved balance, strength, flexibility, mobility, posture, physical confidence and sleep, and pain reduction from conditions such as polymyalgia and osteoarthritis:

*I’d been less flexible and I’ve certainly found the yoga has helped with that. And it’s also helped build up my posture and strength, core strength. So it’s been good for that. I can say that my balance definitely improved, and that was one of the main things I needed to do…. some of the deep breathing has helped me get to sleep at night…. I think my blood pressure had reduced. I think I’d lost a little bit of weight and I was more committed to doing a little bit more exercise. (female, 63)*

The program design was highly valued for supporting individualised age-appropriate practise, encouraging progression and building physical literacy:

*It was an age related program.... so [our instructor] was very open to people who had limitations. She could then show them other ways to do what they’d thought they couldn’t do. It was a lot about changing our thinking process. (female, 80)*

However, not everyone who enrolled in SAGE found it beneficial. Some who had withdrawn from the trial described insurmountable injuries or other health problems which they felt the program could not assist with and, in one case, was exacerbating:

*I had a knee replacement four years ago and the other knee’s got arthritis in it. To start with I would do the class and then I’d think, “Oh dear, my knees are feeling sore”.… But I just kept thinking, “You know what, the more I do it, the better it’s going to get. It’s because I’m not used to doing this type of exercise”. But it didn’t get any better … then I contacted [our instructor] and said, “Look… my knees are really sore and I’m wondering whether I should pull out of the class.“ So she said, “I will modify the exercises for you” which she did…. and I continued to try it for a few weeks, but it still wasn’t getting any better…. So I thought,.. “I’ve just got to stop this”. (female, 75)*

Value expectancy was also affected by the online format: both positively and negatively. The most problematic aspect was reduced visibility, followed by lack of hands-on correction. In phase one of the process evaluation, around half the interviewees who attended the hybrid program felt that online classes were inferior to studio-based classes because their yoga instructor could not observe and correct them effectively over Zoom. This concern was less prominent in the teleyoga participants we spoke to, but a few did speculate that observation and correction in online yoga was poorer:

*I may have struggled with Zoom in making sure things were where they should be. Whilst [our yoga instructor] was terrific, and she would correct you, I think a live [studio] class would be better for that than a Zoom class, because they can actually come up and show you, and move you to where you should be. (female, 62)*

The online format also affected social dynamics. One person who withdrew from the trial explained that teleyoga offered none of the enjoyment she had experienced in the past attending studio classes where there was conversation and laughter with her peers and the instructor:

*Maybe I’m used to more personal interaction, but …. I just found it, yeah, not satisfying doing it online. I felt I wasn’t getting the personal feedback for me. And I found it a bit isolating…. it didn’t feel real … it felt like pretend. (female, 74)*

Positive aspects of the online format that increased the perceived value of SAGE are explored in relation to subsequent program theories below.

### In expert hands

Unsurprisingly, excellent instruction was regarded as essential to participants’ engagement with and confidence in online classes. Qualities identified in the previous phase of process evaluation—such as credibility, empathy, encouragement and the ability to safely lead a whole class while simultaneously attending to the needs of individuals—were equally important in the teleyoga program, partly because they addressed some concerns about reduced visibility outlined above:

*[Our instructor] was brilliant … a fabulous tutor…. I was really intrigued how it would work online because there’s this little screen yet you are doing yoga and a lot of it is being in the right alignment and position. But the way it was set up … you could do it with your eyes closed because she knew exactly what you were doing and would just say, “Gloria, straighten, bend, or lift your shoulder”….. So it was really, really positive to have that attention.… She looked after everybody really, really well. (female, 64)*

However, excellent teleyoga instruction required some additional specialist skills. Instructors had learnt to be more precise in their directions, using their voice as an instrument more consciously. They would orientate themselves and participants towards the camera, spending more time demonstrating poses and making adjustments to maximise visibility:

*… it was helpful for me to be able to see the instructor up close …. I appreciate that she would stop and ask us to watch her so I could bring my iPad right up close and I could see her clearly. (female, 68)*

Adaptability was a core teaching skill in the SAGE program given that it is designed to support individualised practise, but instructors continued to build on this in the online environment, using resources creatively and modifying poses, for example, so that participants could balance using chairs of different heights or practise in constrained spaces:

*I’ve got a couple of people who just work between their bed and their wardrobe, so there’s only one position they can be in and it’s up to me to try and get the best out of them…. It’s made me a better teacher, made me adapt. (Instructor 1)*

The online platform reduced opportunities for interactivity, making it harder for instructors to connect with participants and demonstrate their commitment to building caring and respectful relationships (therapeutic alliances). However, when the program moved online, SAGE introduced brief 1:1 sessions between instructors and each participant prior to starting classes which were valued highly by participants and instructors for generating rapport and confidence that instructors understood and would be responsive to people’s needs:

*… because we did the individual interviews … I was able to really zone in … find out their issues and … whatever they’re dealing with. There’s a sense of creating a space of safety, of care, and that whatever their injuries are I make a note. So I say, “Okay, we can work with that”…. that’s vital…. They’ve got to feel safe with me, they’ve got to feel cared for. (Instructor 3)*

Participants especially appreciated individualised feedback during classes which assured them they were being observed by the instructor and supported to practise effectively:

*She noticed when I wasn’t doing it properly … It was good she pointed it out because I was doing a couple of sessions on my own and I thought I was doing it correctly. If I hadn’t had that interaction, I probably would’ve continued on doing it in my own merry little way, not getting the full benefits of it. (male, 78)*

### A communal experience

Although teleyoga classes were not able to promote social connectivity to the same extent as studio classes, participants did enjoy the shared experience and collegiality of practising with a regular group:

*… being in my own home and with other people in their own home it was a very … friendly atmosphere even though we weren’t together in the same room…. It was a nice feeling that you were with the group, but individual” (female, 79)*

The friendly atmosphere mentioned in this participant quote was something the instructors worked hard to produce. Strategies included instructor-facilitated pre-class chat sessions, using humour and informal language during the class, and encouraging questions and comments:

*I quite liked the informal chats before and after the class. There was a nice sense of where people were at and what they were doing, and the teacher would sometimes participate in that as well, which was really nice…. I quite liked that sense of community that was created with that. (female, 61)*

### Finding yoga within reach

Teleyoga offered greater accessibility and convenience than studio-based classes. People were able to attend (with no commuting) from diverse locations and in a wide variety of circumstances, including during COVID-19 lockdowns and other restrictions. For some, this convenience made the difference between enrolling or not, *“I wouldn’t have joined if it wasn’t online” (female, 80).* Free classes increased accessibility which encouraged waverers to give the program a go and to stick with it in the longer term:

*I was a bit frightened that I wouldn’t be able to do it, but I found that I could. I think if it hadn’t been given to me the way it was – the free, the online, the accessibility – I wouldn’t have done it. (female, 68)*

*… when I saw that it was free of charge, it was an opportunity that I jumped at… If you’re not familiar with yoga, you might need a free-of-charge period to ‘taste and see’ whether it’s for you. (male, 65)*

The gratitude that participants expressed about the hybrid program was also expressed by those who attended teleyoga program and may have been enhanced by the increased accessibility of classes outlined above, especially during the COVID-19-pandemic:

*I’m very grateful to SAGE because it has given a dimension to my life that wasn’t there before. (female, 80)*

While videoconferencing technology increased accessibility in many ways, it also presented some access challenges. Lack of familiarity with the Zoom platform by many participants and the yoga instructors required considerable support from the SAGE team who offered IT coaching and setup advice, including ‘lurking’ in early classes to step in and provide support when needed.

Participants used various devices for online classes including phones, tablets and laptops. Surprisingly, smaller screen size did not seem to be a consideration as those who used phones said they were satisfied with this and only two online participants reported difficulty seeing their instructor, “She seemed a little bit distant for some of the poses but… it wasn’t something that particularly disturbed me” (male, 65). Concerns about restricted lines of sight appeared to be entangled with anxiety about using technology suboptimally rather than screen size:

*I wondered sometimes if I was actually doing the poses correctly … because I couldn’t always see the whole of her and she couldn’t always see the whole of me…. It was just the setup, I didn’t perhaps have the best setup … (female, 69)*

This suggests that improved technical proficiency might reduce concerns about line-of-sight. To an extent, this was tackled in the 1:1 pre-program meetings in which instructors gave advice and helped participants to trial the setup they would use in classes:

*[Our instructor] spent a considerable time getting the setup right for both her and for me, and I think she did it with everybody else…. it made a big difference because I wouldn’t have known where I needed to be …so I had to move around and eventually got it and I was happy with it. (female, 80)*

### Building yoga habits

The four mechanisms identified in this program theory—purposeful structure, momentum, accountability and continuity—were all retained in the teleyoga program which kept the structure of twice-weekly classes over 12 months with progressive challenges. As with the hybrid program, participants found this *“habit forming”* (male, 78):

*It gave you a sense of purpose every week to know or twice a week that you would commit to this. And I just organised … my life around that, knowing that on Monday and Wednesdays, I had yoga at whatever time it was, and I miss that. It’s not the same if you just set aside that time yourself and do your own independent practise (female, 63)*

Similarly, teleyoga participants felt accountable to their instructor and, to an extent, their peers which encouraged attendance and possibly increased determination to make progress:

*[what helped was] just that it was a regular thing that you did twice a week, you had to be prepared for it and.…when it started off, I was prepared to just do it sitting on a chair and then I saw the other people not doing it in a chair, I thought “Well, Joyce, get off your chair and do it properly!”…. Yeah I felt guilty if I didn’t attend the class because I knew the teacher was putting herself forward and the rest of the group was too. (female, 79)*

Participants emphasised the value of synchronous classes rather than pre-recorded videos both in terms of responsive instruction and the sense of commitment to dedicated practise time that live classes generate:

*I always feel time pressure when I’m doing it myself…. You negotiate trying to reduce time. Whereas when you’ve got an online class you say, “Okay, I’m just going to focus on this for an hour”…. If it’s a live event, then you’re committing at a particular time with a particular person or group. (male, 65)*

However, a few participants used the SAGE homework videos to maintain the program structure over breaks and to sustain yoga practice after the program had finished, thus continuity was enhanced by the greater accessibility of teleyoga but also the availability of tailored resources:

*…I’ll continue as I’ve got the videos which are amazing because when [the instructor]’s been away or over the Christmas holidays I’m able to do regular yoga in the morning and it makes such a difference to your day…. Because that helps you get your routine. See, I’m a great believer in rhythm and routine in life … with the video it’s really easy to get that rhythm happening. Without it, you think “I can’t be bothered”. (female, 80)*

### Yoga’s special properties

The two mechanisms identified in this program theory—mindfulness and embodiment^1^—were retained in the teleyoga program and, for some, may have been enhanced. Online participants welcomed yoga’s holistic attention to mind and body, and the combination of physical challenge, breathwork and relaxation which made yoga practice as much about the journey as the destination:

*I’ve always had an aversion to going to gyms and things like that…. But what we did in yoga … was a positive thing. It wasn’t just dealing with getting my muscles to work better, but it was more holistic like working in the garden where ... it’s almost like the activity is part of something else rather than just being an end in itself. (male, 78)*

*It was really surprisingly calming.… You do come with a different mindset than going to the gym or something like that.… it can open another door in your day-to-day living rather than just using it as a sport. It’s just got a different level. It’s the mindfulness…. it’s just how you appreciate your day … so it can be a way of living. (female, 64)*

There was a suggestion that mindfulness could be enhanced in teleyoga classes due to the solitude of practice:

*The mindfulness gave me a peaceful start to my day. I enjoyed knowing that … it was just going to be the teacher talking and me just sitting in the stillness of it all. I yearned for that…. The peace of it. (female, 68)*

A few participants who had previous experience of yoga felt that SAGE delivered *“mindfulness light” (male, 63)* as classes focused on strength, stretching, flexibility and relaxation rather than deeper meditational techniques. But this was regarded as a feature of the SAGE program design rather than the result of online delivery.

### Unique benefits of online classes

One unanticipated finding was that some participants who had signed up for teleyoga said they would not have attended a studio-based class because they felt too self-conscious. So while reduced visibility online was a concern for people who wanted optimal observation and instruction, it was not only a bonus for some others, it was actually a deal-maker:

*I’m a little bit reserved, I don’t know that I would have gone to a studio. I would have felt a bit embarrassed. But this way I didn’t feel embarrassed because I couldn’t see other people looking at me, I just knew it was just the teacher, and that gave me a lot of courage to have a go. (female, 68)*

In addition to the benefit of reduced ‘exposure’, some participants explained that limited visibility of their peers made group practise less distracting, reduced feelings of competition and facilitated greater inward attention and embodiment:

*… having done classes in the past in a live [face-to-face] situation rather than online, I did notice that difference that online you’re not comparing yourself to others, and I think that’s actually helpful because you just focus on what you can do and focus on what it is you’re trying to achieve (female, 63)*

## Discussion

In our previous process evaluation of the SAGE classes that began in yoga studios then moved online, we reported that many participants found online classes less satisfying and effective compared to studio-based classes. They valued the hybrid program because it enabled them to establish relationships with peers and their instructor while in the studio, and to embed yoga skills that provided a foundation for practising remotely. Thus initial studio-based classes mitigated the impact of decreased interaction, observation and precise correction in online classes [51]. This suggested that hybrid programs may be superior to a program offered entirely online, i.e. teleyoga. However, in our current study with participants who enrolled in and attended a teleyoga program, we found that lack of interaction was barely mentioned, and concerns about barriers to observation or correction were less prominent. This suggests that the preference for a hybrid program was, in part, created by the comparison of delivery modes because studio-based classes set expectations about interactivity, observation and correction which all work differently online.

Indeed, the teleyoga program retained most of the positive aspects identified in the hybrid program and was actually more appealing to most of the participants in this study. For some, its benefits were a game-changer as it provided access to classes that would have been impossible to attend in person, and offered an environment with reduced exposure to peer scrutiny which attracted participants who would not have attended studio-based classes due to embarrassment. This is a serious yet underdiscussed issue in designing physical activity programs and environments. Body-related anxiety may be especially powerful for younger people [56], and does seem to reduce with age [57], but middle-aged and older adults have also been found to avoid physical activity at gyms, swimming pools and other public sites due to embarrassment about physical characteristics and/or capabilities, across many countries and cultures [58–60]. Feelings of shame and anxiety about loss of dignity and negative social comparisons may be especially prominent in people who are overweight [61, 62], are living with a disability [63] or are otherwise socially disadvantaged [64]. Online forums can increase psychological and social safety by reducing body exposure [65] and some participants also told us that online classes reduced distractions and competitiveness. This contrasted with feedback from hybrid program attendees who said they enjoyed the superior visibility in studio classes because they liked to benchmark or compare their physical functioning against their peers, suggesting that online classes may better suit people who are non-competitive. Together, these online conditions may be more effective in facilitating mindfulness and embodiment: key mechanisms for engagement with and satisfaction in yoga.

A desire to avoid unfavourable comparison to ideal body types may be one reason why classes designed exclusively for older people were welcomed by our participants. Other reasons identified in our research were that targeted classes are regarded as offering safer, achievable and more effectively tailored exercise with a community of peers. This aligns with studies of older people’s engagement with tailored physical activity programs [66–68] and may also explain why older instructors are often preferred [69].

Screen size did not seem to be a major consideration for SAGE participants in either program format. While this might be expected in the hybrid program where participants were able to observe the instructor and hone poses early on—laying the foundation for later practice—it is more surprising in the online program. This may be in part because of the strategies instructors developed for maximising visibility (including asking participants to set their device to ‘speaker mode’ so the instructor dominated the screen) and improved verbal instruction. It also aligns with participants’ emphasis on *being observed*, rather than *observing*, as their primary consideration for safe and effective yoga practice.

It is important to note that in-person yoga programs do not directly translate to online platforms: they demand additional strategies for optimising safety, effectiveness and participant enjoyment. In our trial, these included structural and resourcing changes to the SAGE yoga program, for example, brief one-to-one Zoom meetings before program commencement and technological support from the research team. Considerable adaption to yoga instruction was also required in SAGE, including techniques for building understanding and rapport between participants and instructors. Warmth in how older people are welcomed to physical activity programs, as well as assistance in easing them into this new territory, have both been identified as mechanisms in enhancing engagement [64]. It is vital that instructors maximise therapeutic alliance (or therapeutic presence [70]) in online forums [30, 71].

Additional strategies for support and engagement might include providing participants with a setup ‘tip sheet’, being explicit about the challenges older people have faced since the pandemic began and advocating self-care, building in feedback mechanisms to inform instructors’ continual learning, and incorporating empowerment-focused coaching techniques in the one-to-one meetings which engage participants in solution-focused planning to address any potential barriers to program adherence [72–75]. A forthcoming paper will collate lessons from the SAGE yoga instructors’ steep learning curve in adapting to online teaching, including detailing the strategies they found worked most effectively.

### Strengths and weaknesses

Our iterative process evaluation is a strength as it builds on rigorous program theory development using realist methods to understand the impact of different mediums on program implementation and engagement [76]. Purposive sampling ensured diverse recruitment; however, this neglected some important groups due to likely self-selection by participants. For example, older people who had insufficient access to or confidence in using digital devices, or unsuitable home environments, or feared that yoga would exacerbate painful conditions would not have enrolled in the trial. This impedes our understanding of how to better support people who are wrestling with these challenges. In particular, we need to learn more about how to provide effective yoga for people with injuries and conditions causing chronic pain as they are a significant proportion of the older demographic who are at most risk of falling. Further research to identify poses and online teaching techniques that are most effective in reducing pain would be beneficial [30]. Lastly, the low response from withdrawn participants meant that we only heard from a few people who left the program due to illness or injury so we still have much to learn about how to broaden the appeal of SAGE for people who we failed to engage for other reasons.

## Conclusion

Teleyoga cannot facilitate hands-on correction or the same quality of observation or interaction as studio-based yoga classes, but there are manageable strategies for optimising visibility, instruction, social connection and therapeutic alliance, and for adapting to constrained home environments, which satisfied most of our participants. Teleyoga increases accessibility (physical and financial), opening it up to people in diverse locations and circumstances; it provides a psychologically safer space which combats self-consciousness and unwanted competitiveness; it may enhance embodiment and mindfulness for some; and it has the potential to be offered relatively cheaply at scale which could support free or reduced price classes for people on low incomes and pensions, thereby encouraging a wider population to try yoga and maintain their practise. While some of our process evaluation participants preferred studio classes, most firmly agreed that teleyoga was much better than no yoga at all. Therefore, in terms of scalable costs, reach, acceptability and sustainability – teleyoga may be the way forward both for fall prevention and for general health and wellbeing.

## Electronic supplementary material

Below is the link to the electronic supplementary material.

**Supplementary Material 1**

**Supplementary Material 2**

**Supplementary Material 3**

## Data Availability

All relevant data are included in the manuscript.

## Footnotes

1 Embodiment is a feeling of being deeply within one’s body rather than viewing and working on it as an object, often involving a state of absorption or flow. This can facilitate caring attentiveness to the body, physical empowerment and physical literacy [55].

## Abbreviations

SAGE: Successful AGEing.
