# Supplementary material for: “I wouldn’t have joined if it wasn’t online”: understanding older people’s engagement with teleyoga classes for fall prevention
Source: BMC Complement Med Ther. 2022 Nov 2;22:283. doi: 10.1186/s12906-022-03756-1 (PMC9628174; doi:10.1186/s12906-022-03756-1)
Supplement: Supplementary file 1 — Supplementary Material 1 [file 12906_2022_3756_MOESM1_ESM.pdf]

Additional file 1.

FOCUS GROUP GUIDE for participants who completed SAGE online

INTRODUCTION
Welcome everyone. Thank you so much for giving us your time today. First of all I just want to let you know I'm recording this session so we capture the information you give us accurately. Is that OK? Great.
START RECORDING

So as you all know, my name is Heidi Gilchrist, I am a postdoctoral research associate at the Institute for Musculoskeletal Health at the University of Sydney, which is the Institute to which most of the leaders of the SAGE program belong.

I haven't been involved in the set up or running of the trial, but rather have been asked by the organisers, my colleagues, to conduct an evaluation of the SAGE trial, which mostly involves interviewing participants who completed or also didn't complete, the SAGE program. I am also a physiotherapist and outside of work I am an intermittent practitioner of yoga so have some understanding of what you have been doing. Now I will ask my colleague Abby to introduce herself, and then if each of you can tell us your name and where you are joining us from today, before we get started on the questions…. Thanks everyone. It's really great to have you here.

Please keep your cameras on so we can see each other, but mute if there's any background noise where you are.

As you know we're meeting today as part of our evaluation of the SAGE yoga program that you took part in. We hope that learning more about your experience will help us and other researchers design better programs like this in the future.

We'll ask you some questions about your experience of the program, and also share some of the ideas we have about it to get your feedback on them. As you probably know, there are no right or wrong answers in a focus group. You've been invited today because you have in-depth knowledge of what it's like to take part in this program, so your personal opinions are very valuable to us. We want to know your views even if they're different to everyone else's. In fact, *especially* if they're different to everyone else's! We need to know about the whole range of views so that we don't miss anything important. We've got people here from different classes so there may be some differences in the style of the class you attended.

We'll move around the group so that everyone has a say but I may not ask everyone to contribute to every question. We ask that you are respectful of each other's views, and each other's privacy, so that means if you talk about this group with anyone outside, you can tell them what was said, but don't name anyone.

Does that sound OK?

PART A. Main focus group questions

1. Thinking back to when you first heard about the yoga program, why did you decide to take part? What were you hoping to get out of it? *[Prompt: Were falls on your radar?]*

2. Let's talk about your experience of the program overall. For example, what would you say to a friend if they asked you about it? *[Prompts: What was good about it? What could have made it better for you?]*

3. We're keen to know if you think anything changed for you physically or mentally because of taking part in SAGE and, if so, what it was about the program that helped you make those changes?

4. We’re particularly interested in how yoga instruction works online: what helps and what could be improved. Did you notice your yoga instructor doing anything that helped? Is there anything they could have done better? Were there challenges? Any benefits to being online rather than in a studio?

PART B. Testing program theories

We have six theories about what makes these yoga classes work for most people. I am going to get Abby to share her screen with you so you can see each theory as I list them and we can get your views on them.

OK, so we think:

1. People enroll in and stick with the program because they anticipate health benefits and feel actual health benefits early on

2. Another reason people value the program, is the quality of yoga instructors: the way they relate to their students, take account of their different needs and support them to achieve the best they can for their capabilities

3. Doing yoga in a group gives a kind of communal experience which makes the classes feel more meaningful than if you were just following a video online

4. Accessibility and convenience of online classes adds value. Do you think this would be different if there weren’t a pandemic?

5. The structure of SAGE (twice weekly classes over 40 weeks plus homework resources) helps build yoga habits because it creates a sense of momentum and purpose, and there’s some accountability too.

6. People engage with yoga classes differently to other forms of physical activity. We think this is because yoga is holistic and combines physical movement with mindfulness and caring attention to the body. *[Prompt: Does that resonate with you?]*

PART C. Final thoughts

5. Do you have any ideas about what might help people keep going with yoga after the program finishes?

6. Lastly, is there anything else you can tell us that might help us to improve programs that aim to support older people to be as active as possible and avoid falls?

Thank you so much for helping us with our research. It’s been really helpful to hear about your experiences.
