# Supplementary material for: “I wouldn’t have joined if it wasn’t online”: understanding older people’s engagement with teleyoga classes for fall prevention
Source: BMC Complement Med Ther. 2022 Nov 2;22:283. doi: 10.1186/s12906-022-03756-1 (PMC9628174; doi:10.1186/s12906-022-03756-1)
Supplement: Supplementary file 2 — Supplementary Material 2 [file 12906_2022_3756_MOESM2_ESM.pdf]

Additional file 2.

INTERVIEW GUIDE for participants who withdrew from SAGE online

Thank you for agreeing to take part in this interview. As you know, we're evaluating the yoga program that you started. For researchers, learning about what *doesn't* work is just as important as learning about what does work, so it's extremely valuable for us to hear from people who didn't complete the program. The information you give us will help us to design better programs in the future that work for more people.

Just to remind you, our conversation today is confidential which means that when we talk about or publish the results of this evaluation we won't use any information that reveals your identity. And before we start, I want to let you know that I'm **audio recording** our conversation so that I capture our conversation accurately. Is that OK? Thank you.

Part A. Reasons for participation

1. We're interested in why people sign up for program like this. Thinking back to when you first heard about the yoga program, what made you decide to take part? What were you hoping to get out of it?

2. What were your views about yoga at the time?

3. What about falls – was that something you had been thinking about?

4. Did you have any concerns about taking part in the program at that stage?

Part B. Experiences of participation

5. Thinking back to the yoga class(es) you attended, can you talk me through your experience of that? What was it like? I'm keen to hear everything you remember. *[Probe: Was it different from what you expected? Did you notice your feelings change at any point?]*

6. How did you reach the decision to stop yoga classes? →

Probe for problems with:

- *yoga itself*
- *the instructor*
- *program structure/frequency*
- *use of technology*
- *effectiveness of teleyoga*
- *health problems / pain and why yoga didn't fit with their recouperation plan*

7. Is there anything that we could have done that might have helped you stay with the program? *[Prompts: Perhaps a different program structure, or something your instructor could have done differently?]*

Part C. Impacts and maintenance

8. *[Only if they completed at least 3 weeks of the program]* Do you think anything has changed for you physically or mentally because of taking part in this yoga program?

9. Would you consider doing a different sort of program or another form of exercise to gain better strength and balance? What would that look like? Are you currently doing anything like that?

10. Lastly, do you have any advice for people like us who are looking for ways to help older people stay healthy and active, and avoid falls?

Thank you so much for helping us with our research. It's been really helpful to hear about your experience.
